# Incidence of Influenza A(H1N1)pdm09 Infection, United Kingdom, 2009–2011

**DOI:** 10.3201/eid1911.130295

**Published:** 2013-11

**Authors:** Saranya Sridhar, Shaima Begom, Alison Bermingham, Katja Hoschler, Walt Adamson, William Carman, Maria D. Van Kerkhove, Ajit Lalvani

**Affiliations:** Imperial College London, London, UK (S. Sridhar, S. Begom, M.D. Van Kerkhove, A. Lalvani);; Public Health England, Colindale, UK (A. Bermingham, K. Hoschler);; West of Scotland Specialist Virology Centre, Glasgow, Scotland, UK (W. Adamson, W. Carman)

**Keywords:** human influenza, natural infection, pandemics, cohort studies, epidemiology, United Kingdom, England, adult, influenza A(H1N1)pdm09, pandemic influenza, seroprevalence, viruses, seroepidemiology, incidence

## Abstract

We conducted a longitudinal community cohort study of healthy adults in the UK. We found significantly higher incidence of influenza A(H1N1)pdm09 infection in 2010–11 than in 2009–10, a substantial proportion of subclinical infection, and higher risk for infection during 2010–11 among persons with lower preinfection antibody titers.

Case-based population-level surveillance and cross-sectional serologic surveys to estimate incidence and patterns of influenza infection are limited by the lack of accurate denominator data, inability to account for subclinical infections, difficulties in distinguishing between antibodies induced by natural infection and vaccination, and use of samples from high-risk groups. For these reasons, community-based longitudinal studies are ideal to estimate the incidence of infection and spectrum of illness. However, studies of this design describing the 2009 pandemic of influenza A(H1N1)pdm09, reported only from Hong Kong, Singapore, and Vietnam, examine only the 2009–10 season (*1*–*3*).

The epidemiology of A(H1N1)pdm09 in the United Kingdom during 2009–2011 was characterized by 3 distinct waves: first wave, April–August 2009; second wave, September 2009–April 2010; and third wave, August 2010–April 2011. We report results from a community-based longitudinal cohort study that compared the epidemiology of influenza A(H1N1)pdm09 infection over the second and third waves. The North West London Research Ethics Committee approved this study (reference 09/H0724/27).

## The Study

**Figure 1:**
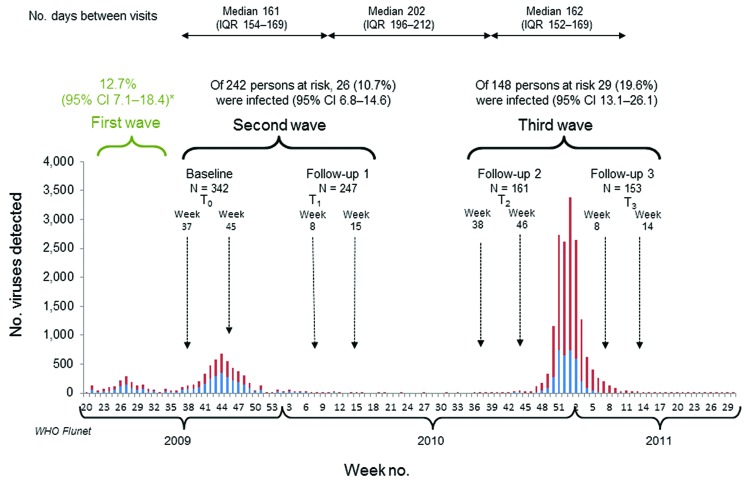
Incidence of natural influenza A(H1N1)pdm09 infection in the study cohort during the 3 pandemic waves in context of the evolving pandemic, United Kingdom. Study outline is depicted in the upper panel in temporal context of the pandemic during the 2009–2011 influenza seasons. The bar chart shows UK influenza virologic surveillance data from WHO Flunet (www.who.int/influenza/gisrs_laboratory/flunet/en/) highlighting the periods of study recruitment and follow-up in relation to influenza A activity in the United Kingdom during 2009–2011. Red bars indicate influenza A of all subtypes; blue bars indicate the number of A(H1N1)pdm09 cases detected by virologic national surveillance. Healthy adults were recruited after the first pandemic wave (April–August 2009) had ended in the United Kingdom and were followed over 2 influenza seasons, with serum samples collected before and at the end of each influenza season. The median time between visits is shown. The second wave was defined as baseline (September–November 2009) to first follow-up (February–April 2010) and the third wave as the time between the second follow-up (August–November 2010) and the third follow-up (February–April 2011). The green bracket and numerals represent the estimated cumulative incidence of infection over the first pandemic wave by calculating the difference between and seroprevalence rates at baseline in the cohort and prepandemic (2008) published seroprevalence rates. Infection was defined as detection of A(H1N1)pdm09 virus in nasal swabs returned during the second or third wave or a 4-fold rise in A(H1N1)pdm09 virus HI titer in paired serum samples collected at the start and end of each wave. The number of infected persons with total persons at risk during each of the second and third waves with calculated incidence rate and 95% CIs are shown. WHO, World Health Organization; IQR, interquartile range; HI, hemagglutination-inhibition. *Infection rates in the first wave reflect cumulative incidence of infection, estimated by calculating the difference in proportion of persons with HI titer >32 between baseline (T_0_) and published Health Protection Agency data before the pandemic in 2008.

A total of 342 healthy adult staff and students of Imperial College London (London, UK) were recruited during September–November 2009 and followed for 2 consecutive influenza seasons: 2009–10 and 2010–11 (Figure 1). Participants’ median age was 28 years (interquartile range 20–36 years); 83% were <40 years of age. At each time point, collected serum samples were tested for antibodies to A(H1N1)pdm09 virus (A/England/195/09 strain) by the hemagglutination-inhibition (HI) assay (*4*). Participants were asked to record temperature, self-sample, and return nasal swabs when experiencing influenza-like symptoms. Swabs were tested for respiratory viruses with standardized real-time reverse transcription PCR. Influenza seroprevalence rates were defined as the proportion of persons with HI titers >32 (*4*).

Because our study began at the end of the first pandemic wave, cumulative incidence of A(H1N1)pdm09 infection over the first wave was estimated as the difference between age-specific seroprevalence rates at recruitment (T_0_ in Figure 1) and published prepandemic (2008) seroprevalence rates for England (*4*). Incident infection was defined as antibody seroconversion (4-fold rise in HI titer) in paired serum samples collected at the start and end of a wave among unvaccinated persons (because HI assay cannot differentiate infection from vaccination) or detection of A(H1N1)pdm09 virus in nasal swabs. The incidence of infection was estimated for the second and third waves as the proportion of incident infections among unvaccinated participants.

Development of any symptoms was recorded on a Web-based questionnaire emailed to participants every 3 weeks. The average response rate was 75%. Illness episodes were categorized as acute respiratory infection (episode with any symptoms), influenza-like illness ([ILI] episode with fever plus cough or sore throat), and fever (recorded temperature >38°C) alone. Visits to primary care or hospital during illness were also recorded. Data were analyzed using Stata version 9.0 (StataCorp, College Station, TX, USA) with the χ^2^ test to compare proportions and *t* test to compare means after checking for normal distribution by assessing for kurtosis, skewness, and the Shapiro-Wilk test. Hosmer-Lemeshow test was used to estimate goodness-of-fit for each logistic regression.

At recruitment, after the first pandemic wave, A(H1N1)pdm09 seroprevalence was 26% (95% CI 21.4–31.2), with seroprevalence significantly higher in participants 18–25 years of age than in older age groups (Table 1). Participants with ILI in the preceding 3 months corresponding to the first wave had significantly higher (p<0.001) mean A(H1N1)pdm09 virus HI titers, which in conjunction with the age distribution, suggests first-wave infection rather than cross-reactive antibodies (*5*). Overall cumulative incidence during the first wave was 12.7% (95% CI 7.1%–18.4%) and 26.6% (95% CI 15.3%–37.8%) among participants 18–25 years of age with no increase in older age groups (Technical Appendix Table 1).

**Table 1:** Seroprevalence of influenza A(H1N1)pdm09 antibodies at baseline, United Kingdom, 2009–2011*

Risk factor	HI titer, no. (%)†					GMT (95% CI)	p value§
	<8	8–32	>32	Total	p value‡		
Total	202 (62.0)	39 (12.0)	85 (26.1)	326		11.6 (10.0–13.4)	
Sex							
M	92 (58.2)	22 (13.9)	44 (27.8)	158	0.48	12.8 (10.3–15.8)	0.19
F	110 (65.5)	17 (10.1)	41 (24.4)	168		10.6 (8.7–12.8)	
Age group, y¶							
18–25	57 (44.9)	15 (11.8)	55 (43.3)	127	Ref	20.4 (15.5–26.8)	Ref
26–40	99 (73.9)	19 (14.2)	16 (11.9)	134	<0.001	7.8 (6.6–9.1)	<0.001
41–55	32 (74.4)	2 (4.7)	9 (20.9)	43	0.01	8.6 (6.2–11.8)	<0.001
>56	9 (64.3)	1 (7.1)	4 (28.6)	14	0.29	9.2 (5.3–16.0)	0.14
Seasonal influenza vaccination in 2008#							
Yes	23 (54.8)	5 (11.9)	14 (33.3)	42	0.19	12.6 (8.6–18.3)	0.56
No	174 (64.2)	32 (11.8)	65 (24.0)	271		11.1 (9.5–13.0)	
Self-reported history of ILI in 3 mo before recruitment**							
Yes	9 (36.0)	3 (12.0)	13 (52.0)	25	<0.01	35.7 (16.5–77.0)	<0.001
No	189 (64.3)	36 (12.2)	69 (23.5)	294		10.5 (9.2–12.1)	

*HI, hemagglutination Inhibition; GMT, geometric mean titer; Ref, referent; ILI, influenza-like illness. †Of the 342 participants in the study, 16 were missing data on HI assay results. ‡p value comparing the number of persons with a titer >32. §p value comparing the GMT. For age categories, p value represents the test for trend. ¶Data available for 314 persons. #Data available for 313 persons. **Data available for 319 persons.

The incidence of infection over the third pandemic wave was significantly higher (p = 0.02) than over the second wave (Figure 1). Among participants with prewave titers <8, the incidence of infection was significantly higher over the third wave than over the second wave (p<0.001); incidence did not differ for participants with prewave titers >8 (Table 2, Appendix). Age-specific incidence was significantly higher (p = 0.01) over the third wave than the second wave among participants 26–40 years of age (third wave: 25.4% [95% CI 15.2–35.5]; second wave: 10.9% [95% CI 5.1–16.7]) but not the other age groups (Table 2, Appendix). For 11 infected participants with paired serum samples and virus detected in nasal swabs, 2 (18%) did not show antibody seroconversion (Technical Appendix Table 2).

**Table 2:** Risk factors for natural infection with influenza A(H1N1)pdm09, United Kingdom*

Risk factor	Second pandemic wave (Sep 2009–Apr 2010)									Third pandemic wave (Aug 2010–Apr 2011)								
	Infection status, no.(%)			Unadjusted			Adjusted†			Infection status, no. (%)				Unadjusted			Adjusted†	
	None	Natural‡	Total	OR (95% CI)	p value		OR (95% CI)	p value		None	Natural‡	Total		OR (95% CI)	p value		OR (95% CI)	p value
Total	210 (86.4)	26 (10.7)	242							119 (80.4)	28 (19.6)	148						
Sex																		
M	95 (87.2)	14 (12.8)		Ref						53 (85.5)	9 (14.5)	62		Ref				
F	121 (91.9)	12 (9.0)		0.67 (0.30–1.52)	0.34		0.68 (0.30–1.58)	0.37		66 (76.7)	20 (23.3)	86		1.78 (0.75–4.24)	0.19		1.67 (0.69–4.26)	0.28
Age group, y							0.99 (0.95–1.03)	0.6									0.98 (0.93–1.02)	0.29
18-25	75 (91.5)	7 (8.5)	82	Ref	Ref					33 (80.5)	8 (19.5)	41		Ref				
26-40	98 (89.1)	12 (10.9)	110	1.31 (0.49–3.49)	0.59					53 (74.7)	18 (25.4)	71		1.40 (0.55–3.58)	0.48			
41-55	30 (85.7)	5 (14.3)	35	1.79 (0.53–6.07)	0.35					23 (88.5)	3 (11.5)	26		0.54 (0.13–2.25)	0.39			
>56	10 (90.9)	1 (9.1)	11	1.07 (0.12–9.64)	0.95					7 (57.1)	0	7		–	–			
Not known	4 (80.0)	1 (20.0)	5							3 (100.0)	0							
Titer at start of season							0.98 (0.97–1.01)	0.23									0.92 (0.86–0.99)	0.04
<8	140 (88.5)	18 (11.5)	158	Ref	Ref					61 (70.1)	26 (29.9)	87		Ref				
8–32	49 (89.1)	6 (10.9)	55	0.95 (0.36–2.54)	0.92					23 (88.5)	3 (11.5)	26		0.31 (0.08–1.11)	0.07			
>32	27 (93.1)	2 (6.9)	29	0.58 (0.13–2.63)	0.48					35 (100)	0	35		–	–			

*Ref, referent; –, cannot be calculated. **†**Adjusted for the other covariates. In the logistic regression undertaken the dependent variable was infection status and age, sex and titer at the start of the season were the independent variables. The results shown are from a single run, and no other model was fitted. Hosmer Lemeshow goodness-of-fit statistic showed an adequate fit for the logistic regression models fitted to the second wave and the third wave data (Hosmer Lemeshow statistic was 4.69 ,p = 0.58 for the second wave and 5.54, p = 0.59 for the third wave). ‡Natural infection indicates incident infection among those susceptible at the beginning of the wave.

**Figure 2:**
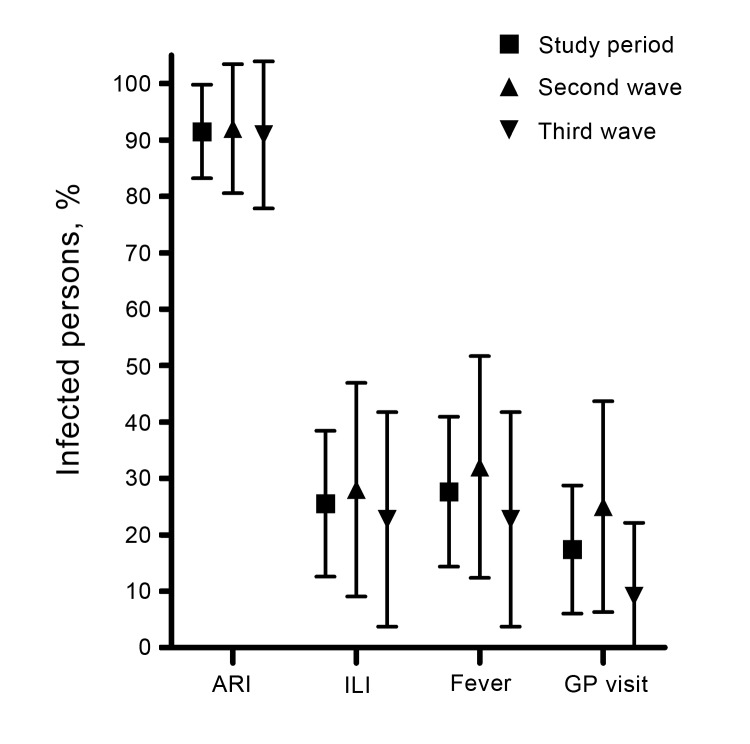
Proportion of influenza A(H1N1)pdm09–infected persons who had symptoms during their illness episode during the second wave (September 2009–April 2010), third wave (August 2010–April 2011), and entire study period, United Kingdom. Proportion of persons with reported symptoms over the study period is combined from the second and third waves. Symptoms were recorded by a Web-based symptom questionnaire emailed to participants every 3 weeks. Symptoms associated with illness episode were acute respiratory infection (ARI; illness episode with any symptoms), influenza-like illness (ILI; episode with fever plus cough or sore throat), fever (recorded temperature >38°C) alone, or visit to a general practitioner (GP). The graph depicts the average with 95% CIs calculated by using binomial distribution.

During an illness episode, 20% of infected participants reported fever or ILI, 17% visited their general practitioner, and none visited a hospital (Figure 2). Because predictions of a small third pandemic wave were disproved (*4*), the reasons for this large wave remained unclear. Multivariate logistic regression was undertaken with infection as the dependent variable and age, sex, and prewave titers as independent variables. Each doubling increase in prewave HI titers, after adjustment for age and sex, was associated with significantly lower risk for infection (odds ratio 0.92, 95% CI 0.9–1.0, p = 0.04) during the third, but not the second, wave (Table 2, Appendix).

## Conclusions

Incidence of A(H1N1)pdm09 infection was significantly higher among healthy adults during the third pandemic wave (2010–11) than during the second wave (2009–10). This study complements and corroborates clinical surveillance data and population-sampling seroepidemiology from the United Kingdom (*4*,*6*,*7*), United States (*8*) and elsewhere (*9*).

The reasons for this unexpectedly larger third wave in the postpandemic season remain unclear. We show an increased risk for A(H1N1)pdm09 infection associated with lower antibody levels at the start of the season, irrespective of age, during the third, but not the second, wave. Because no substantial viral genetic change occurred between the waves (*7*), our finding suggests that the third wave was driven by infection among susceptible persons remaining antibody-naive at the end of the second wave. This thesis is supported by serosurveillance data showing lower infection rates over the third wave among age groups with the highest infection rates over previous pandemic waves (*7*,*8*). Our interpretation is further strengthened by a meta-analysis of serologic data from 19 countries that showed 20%–27% incidence of infection during the first pandemic year, suggestive of a large population susceptible to infection in subsequent seasons (*10*).

Incidence in our cohort was lower than that estimated for England by cross-sectional serosurveys (*7*,*11*). This finding may reflect our accounting for individual-level vaccination status and baseline antibody titers; data usually unobtainable with cross-sectional population-sample serosurveys. However, our study did not include children or elderly persons, which limits the generalizability of our findings. A major advantage of longitudinal cohort studies recording clinical data is identification of subclinical and asymptomatic infections. More than 80% of participants did not seek primary care or have surveillance-defined ILI indicating a high proportion of subclinical infection among healthy adults undetectable by routine case-based surveillance. We also describe persons shedding virus without antibody seroconversion, a phenomenon recently reported in Vietnam and the United Kingdom (*4*,*12*). Although these nonseroconverters might have antibodies detectable by microneutralization assay, such nonseroconverters, undetectable by serosurveys using the standard HI assay, further highlight the possibility of underestimating community infection rates when cross-sectional serosurveys alone are used. 

Despite our intensive symptom ascertainment, 4 participants with influenza reported no symptoms. Cross-reactive cellular immune responses that are highly prevalent in the population (*13*) have recently been shown to be associated with protection against symptomatic illness (*14*). 

Our analysis of pandemic influenza in a community cohort over successive seasons offers insight into contributors of the unexpectedly larger third pandemic wave. Our analysis also highlights the necessity of using cohorts to complement routine case-based surveillance to estimate influenza burden.

Technical Appendix Method of identifying influenza A(H1N1)pdm09 infection during the second and third pandemic waves and cumulative incidence of A(H1N1)pdm09 infection during the first pandemic wave, United Kingdom.
