# Supplementary material for: Incidence of Influenza A(H1N1)pdm09 Infection, United Kingdom, 2009–2011
Source: Emerg Infect Dis. 2013 Nov;19(11):1866–9. doi: 10.3201/eid1911.130295 (PMC3837661; doi:10.3201/eid1911.130295)
Supplement: Technical Appendix — Method of identifying influenza A(H1N1)pdm09 infection during the second and third pandemic waves and cumulative incidence of A(H1N1)pdm09 infection during the first pandemic wave, United Kingdom. [file 13-0295-Techapp-s1.pdf]

Article DOI: http://dx.doi.org/10.3201/eid1911.130295

Incidence of Influenza A(H1N1)pdm09 Infection, United Kingdom, 2009–2011

Technical Appendix

Technical Appendix Table 1. Cumulative incidence of influenza A(H1N1)pdm09 infection during the first pandemic wave (April–August 2009), United Kingdom

	Seroprevalence rates for A(H1N1)pdm09 virus in 2008 from Health Protection Agency data*		Seroprevalence rates at baseline (2009)*		Cumulative Incidence†
Demographic	n/N‡	% (95% CI)	n/N‡	% (95% CI)	Difference between T_0 and 2008 (95% CI)
Overall	66/495	13.33 (10.34–16.32)	85/326	26.07 (21.39–31.19)	12.74 (7.1–18.36)
Age group, y					
18–24	21/120	17.5 (10.70–24.30)	52/118	44.06 (35.11–53.03)	26.56 (15.32–37.81)
25–44	14/158	8.86 (4.43–13.29)	21/158	13.29 (7.99–18.58)	4.43 (–2.47–11.33)
45–64	31/217	14.29 (9.63–18.94)	9/39	23.08 (9.85–3.63)	8.79 (–5.22–22.81)
≥65	128/549	23.32 (19.78–26.85)	2/3	66.66 (13.32–120.01)	43.35 (–10.11–96.81)

*Seroprevalence rates indicate the proportion of persons with hemagglutination-inhibition titer to pdmH1N109 virus >32
†Cumulative incidence was calculated by the difference in proportions between the 2 time points.
‡n = number of persons with HI titer >32; N = total number of persons in cohort tested.

Technical Appendix Table 2. Method of identifying influenza A(H1N1)pdm09 infection during the second and third pandemic waves, United Kingdom

	Second wave (Sep 2009–Apr 2010)*			Third wave (Aug 2010–Apr 2011)*		
Variable	No. positive	Total no. samples	%	No. positive	Total no. samples	%
Total A(H1N1)pdm09 infected (RT-PCR positive and/or seroconversion)	26	242	10.74	29	148	19.59
Nasal swab RT-PCR positive	6	48	12.50	7	45	15.56
Seroconversion (4-fold rise in antibody)	24	242	9.92	27	146	18.49
Seroconversion and RT-PCR positive	4	39	10.26	5	39	12.82

*One person with a positive nasal swab did not show seroconversion to A(H1N1)pdm09 virus, and 1 did not have a paired serum sample to measure antibody seroconversion. RT-PCR, reverse transcription PCR.
